# Tightening Up the Nomenclature for Non-Physician Clinicians: Why Not Call All of Them Physician Assistants?

**DOI:** 10.9745/GHSP-D-14-00217

**Published:** 2015-03-02

**Authors:** Luppo Kuilman, Gomathi Sundar

**Affiliations:** Hanze University of Applied Sciences, Groningen, Netherlands; Indian Association of Physician Assistants, Chennai, India

Dear Editor-in-Chief,

This Letter is in reference to the article by KD Rao et al., “As good as physicians: patient perceptions of physicians and non-physician clinicians in rural primary health centers in India,” published in the November 2013 edition of *Global Health: Science and Practice* (GHSP).^1^ The authors have to be lauded for their efforts to produce evidence on patient perceptions of care provided by non-physician clinicians (NPCs)—an issue that receives little attention, as most of the focus is on the technical quality of care provided by such clinicians.

Debates have been ongoing about the best course of action to meet the health care needs of rural populations in India.^2^^,^^3^ Given that the Indian health care sector is a vast system with diverse needs and attendant challenges, it is reassuring to know that there are potential viable alternatives to allopathic doctors in areas with physician shortages.

Rao and colleagues introduced GHSP readers to a cadre of health care workers called rural medical assistants (RMAs). We would like to introduce to readers another cadre that has been present for nearly 2 decades in the Indian health care sector and that has been largely ignored for just as long—that of the physician assistant (PA).

The Indian PA workforce consists of close to 1,000 qualified health care professionals who are mainly deployed in hospital settings in urban areas. The PA program as offered by several institutions and their affiliated universities varies in duration, but in general, requires 3 years of didactic medical education followed by a 1-year internship in a chosen medical specialty; graduates receive a Bachelor of Science degree.^4^ Besides taking extensive coursework in basic and medical sciences and theory, PAs receive practical training to perform many medical tasks, including taking patients' medical history, conducting physical examinations, preparing medical notes, writing discharge summaries, counseling patients, and participating in clinical decision making and treatment. They undergo clinical rotations in major medical specialties throughout the program, and their performance is continually assessed.

Globally, the PA profession has its roots in the United States beginning in the early 1960s. As of 2013, the American PA workforce consisted of more than 90,000 certified clinicians, and training was offered at more than 173 programs throughout the country. Most programs grant a master's degree, and graduates are working in all medical specialties including primary care.^5^ Meanwhile, many other countries have observed the success of the PA profession and successively have employed PAs as a way to overcome the imbalance between the demand and supply of medical care, including Australia, Canada, England, Germany, Ghana, The Netherlands, and Scotland.^6^^–^^9^ Other countries are exploring the possibilities of deploying PAs, but clear documentation on their respective successes is still lacking.

Several local initiatives in India are currently employing different types of NPCs. Many look down upon such NPCs and set them aside as “half-doctors.” Understandably, there is concern that some NPCs have little medical education and/or training and are thus not qualified to diagnose and treat patients. But we appeal to all stakeholders involved in planning and managing the Indian health industry to grant a professional identity to those NPCs who are adequately trained. In this context, we feel it is very important to raise this topic to consider standardizing several local NPC initiatives into one new type of medical provider, regardless of whether they practice medicine in (rural and remote) family practices or in hospital settings. As long as the NPCs share more commonalities than differences in terms of educational outcomes and the medical tasks they are expected to perform, it may be plausible to classify them under one professional identity, i.e., as physician assistants. Classifying NPCs under the identity of the PA profession would also make it easier for societies to know whom to trust in a health care worker.

One reason why some countries have been successful in deploying PAs in their health systems may be because the initiatives were government-driven in contrast to the Indian situation. The State Medical University of Tamil Nadu in India has recognized the importance of PAs and has established a PA program in collaboration with a number of hospitals in the state, but, sadly enough, the program is yet to be under the purview of the Ministry of Health and Family Welfare.

Some stakeholders in India want to explore allowing AYUSH practitioners to practice allopathy to address physician shortages.^10^ AYUSH practitioners are trained in Indian systems of medicine (Ayurveda, Yoga, Unani, Siddha, Homeopathy). However, we think it would be more prudent to assist AYUSH practitioners in finding opportunities to practice within their own field and instead to explore the possibility of expanding the success of Indian PAs into rural areas. After all, several studies have demonstrated American PAs working successfully in rural and remote areas, including with respect to high patient satisfaction,^11^ good job satisfaction for the PAs themselves,^12^ and also to the satisfaction of the medical society.^13^

## Notes

**First published online:** 2015 Feb 9
